# Optimizing bowel recovery after laparoscopic donor nephrectomy

**Published:** 2008

**Authors:** Gagan Prakash, Gagan Gautam

**Affiliations:** Department of Urology and Renal Transplant, Fortis Flt. Lt. Rajan Dhall Hospital, Sector B, Pocket 1, Aruna Asaf Ali Marg, Vasant Kunj, New Delhi - 110 070, India. E-mail: gagangg@gmail.com

## SUMMARY

The authors studied the impact of using preoperative bowel preparation and postoperative ketorolac in reducing duration of hospital stay in donors undergoing laparoscopic nephrectomy. Over a five-year period, 300 patients underwent laparoscopic donor nephrectomy (LDN) for renal transplant by a single surgeon. Preoperative evaluation followed standard protocol for all donors. The mean age was 36.7 years (range 18-68); 167 were women and 133 were men. The mean body mass index was 28.3 kg/m^2^ (range 23-34). The mean operative time was 180 +/− 55 min with a mean blood loss of 80 +/− 50 ml and a warm ischemia time of 4 +/− 2 min. All patients underwent strict preoperative bowel preparation and postoperative analgesia regimens. The bowel preparation regimen included two days of clear liquid diet before surgery and two bottles of magnesium citrate orally (150 ml) the day before surgery. The patients self-administered a Fleets enema the evening before surgery and all patients fasted after midnight before surgery. After removal of the kidney, 30 mg of intravenous ketorolac was injected in all donors. Postoperative analgesia regimen included 30 mg of intravenous ketorolac as a bolus every 6 h for a maximal duration of 48 h. The discharge criteria included ability to ambulate, tolerance of a regular diet, absence of nausea, passage of flatus, stable vital signs, stable hematocrit and adequate pain management with oral medications. The patients in whom this regimen was followed had a mean hospital stay of 1.1 days and tolerated a clear liquid diet for breakfast and regular diet for lunch on the first postoperative day. Based on these findings the authors concluded that a thorough preoperative bowel preparation coupled with postoperative ketorolac intravenous analgesia resulted in an earlier bowel recovery in patients undergoing LDN thereby permitting an earlier discharge from hospital.

## COMMENTS

Postoperative recovery and duration of hospital stay has always been a concern for the surgeon, the patient and the hospital authorities. It assumes even more importance if the patient is a prospective healthy donor for organ transplantation.

The establishment of LDN as a standard procedure of choice has contributed a lot towards reducing donor morbidity. Measures to minimize it further are always welcome.

Recovery from postoperative ileus with resumption of normal diet is a major criterion for discharging donors after an LDN. In this study, authors have evaluated the role of preoperative bowel preparation and use of ketorolac as an analgesic in hastening recovery of bowel function. Both animal and human studies have proved that ketorolac helps in recovery of postoperative ileus.[1,2] When administered preoperatively, ketorolac prevents delay in intestinal transit and inhibition of myoelectric activity seen in postoperative ileus. This is due to the effect of ketorolac on intestinal migrating myoelectric activity. Ketorolac does have its side-effects like impairment of renal function and risk of bleeding, however, these have been found to be transient and minor.[3] The authors had already found a beneficial effect of ketorolac in open donor nephrectomy in a previous study.[4] Similar advantage has been found in LDN in the present study.

The second intervention in this series is preoperative bowel preparation. The authors propose that preoperative bowel preparation before LDN has a role in reducing postoperative paralytic ileus. Contrary to conventional thinking, recent studies have disapproved the correlation between bowel preparation and postoperative ileus.[5] Mechanical bowel preparation is now recommended only in selected cases where palpation of the entire colon during surgery or intraoperative colonoscopy might be required.[6] Furthermore, the present study analyzes the impact of two interventions on one outcome i.e. postoperative ileus. This does not allow us to establish that both these factors i.e. ketorolac analgesia and bowel preparation play a role in bowel recovery as it is difficult to comment whether the recovery from postoperative ileus was due to bowel preparation, ketorolac or both. This is not the ideal study design in this situation since the role of each of these variables should be studied independently to establish a cause and effect relationship.

With conflicting evidence for the role of bowel preparation in reducing ileus and the limitations of this study, it would probably be unwise to subject healthy donors to a cumbersome bowel preparation regimen starting two days prior to surgery. Larger prospective randomized trials are required to establish bowel preparation as an independent factor hastening recovery from ileus.

Switching over from opioid to ketorolac for postoperative analgesia, however, is worthy of recommendation since the use of opioid analgesics in the postoperative period not only delays the recovery of bowel function but also may result in undue sedation and respiratory depression.
